# 1144. Rate of Infusion Reactions Among Patients Receiving Casirivimab/Imdevimab in the Home Setting

**DOI:** 10.1093/ofid/ofac492.982

**Published:** 2022-12-15

**Authors:** Michelle Simpson, Danell Haines

**Affiliations:** National Home Infusion Association, Peachtree City, Georgia; Haines Consulting, Columbus, Ohio

## Abstract

**Background:**

Monoclonal antibodies used in the treatment and prevention of COVID 19 infection are an emerging area of infectious disease. Casirivimab/imdevimab received emergency use authorization (EUA) for the prophylaxis and treatment of COVID 19 disease. Infusion reactions may occur with the administration of monoclonal antibodies and can be relieved by slowing or stopping the infusion rate. During the casirivimab/imdevimab EUA, the National Home Infusion Foundation (NHIF) collected data to determine patient outcomes and the incidence of infusion reactions. Infusion rate and premedication protocols were also studied.

**Methods:**

Home infusion companies nationwide were invited to participate in this study by completing a short survey to determine eligibility. The data variables investigated included infusion time, adverse events, and whether standard orders for premedications were used. The data was collected using an Excel® spreadsheet and a follow-up survey verified the relationship between the length of infusion and ADR incidence. The data was imported to IBM SPSS® (Statistical Product and Service Solutions®) for additional analysis.

**Results:**

With this patient sample, the infusion time was either 20, 30, or 50 minutes with most (62.60%) being 20 minutes (Exhibit: Infusion Time). Infusion rates were based on organization protocols, standard prescriber orders, or the clinical needs of the patient. Of the 464 patient cases, 95.26% (n=442) had no reported adverse event. Of the 22 cases with a reported event, the most common symptoms were fever (6) and hypotension (4). Premedications were not routinely included in standard prescribing orders and were based on patient specific situations.

**Conclusion:**

Administration of casirivmab/imdevimab in the home setting showed a low incidence of adverse drug reactions, and the incidence of infusion reactions were not directly related to infusion time or premedication use.

**Disclosures:**

**All Authors**: No reported disclosures.

